# Integrative Effect of Carvedilol and Aerobic Exercise Training Therapies on Improving Cardiac Contractility and Remodeling in Heart Failure Mice

**DOI:** 10.1371/journal.pone.0062452

**Published:** 2013-05-01

**Authors:** Andréa S. Vanzelli, Alessandra Medeiros, Natale Rolim, Jan B. Bartholomeu, Telma F. Cunha, Luiz G. Bechara, Enéas R. M. Gomes, Katt C. Mattos, Raquel Sirvente, Vera Salemi, Charles Mady, Carlos E. Negrao, Silvia Guatimosim, Patricia C. Brum

**Affiliations:** 1 School of Physical Education and Sport, University of São Paulo, São Paulo, Brazil; 2 Biosciences Department- Federal University of São Paulo, Santos, Brazil; 3 Department of Circulation and Medical Imaging and K.G. Jebsen Center of Exercise in Medicine, Trondheim, Norway; 4 Physiology and Biophysics Department, Federal University of Minas Gerais, Belo Horizonte, Brazil; 5 Heart Institute (InCor), University of São Paulo, Medical School, São Paulo, Brazil; Scuola Superiore Sant’Anna, Italy

## Abstract

The use of β-blockers is mandatory for counteracting heart failure (HF)-induced chronic sympathetic hyperactivity, cardiac dysfunction and remodeling. Importantly, aerobic exercise training, an efficient nonpharmacological therapy to HF, also counteracts sympathetic hyperactivity in HF and improves exercise tolerance and cardiac contractility; the latter associated with changes in cardiac Ca^2+^ handling. This study was undertaken to test whether combined β–blocker and aerobic exercise training would integrate the beneficial effects of isolated therapies on cardiac structure, contractility and cardiomyocyte Ca^2+^ handling in a genetic model of sympathetic hyperactivity-induced HF (α_2A_/α_2C_- adrenergic receptor knockout mice, KO). We used a cohort of 5–7 mo male wild-type (WT) and congenic mice (KO) with C57Bl6/J genetic background randomly assigned into 5 groups: control (WT), saline-treated KO (KOS), exercise trained KO (KOT), carvedilol-treated KO (KOC) and, combined carvedilol-treated and exercise-trained KO (KOCT). Isolated and combined therapies reduced mortality compared with KOS mice. Both KOT and KOCT groups had increased exercise tolerance, while groups receiving carvedilol had increased left ventricular fractional shortening and reduced cardiac collagen volume fraction compared with KOS group. Cellular data confirmed that cardiomyocytes from KOS mice displayed abnormal Ca^2+^ handling. KOT group had increased intracellular peak of Ca^2+^ transient and reduced diastolic Ca^2+^ decay compared with KOS group, while KOC had increased Ca^2+^ decay compared with KOS group. Notably, combined therapies re-established cardiomyocyte Ca^2+^ transient paralleled by increased SERCA2 expression and SERCA2:PLN ratio toward WT levels. Aerobic exercise trained increased the phosphorylation of PLN at Ser^16^ and Thr^17^ residues in both KOT and KOCT groups, but carvedilol treatment reduced lipid peroxidation in KOC and KOCT groups compared with KOS group. The present findings provide evidence that the combination of carvedilol and aerobic exercise training therapies lead to a better integrative outcome than carvedilol or exercise training used in isolation.

## Introduction

Heart failure (HF) is a common endpoint for many forms of cardiovascular disease. In addition, this syndrome is the leading cause of morbidity and mortality in older individuals [1]. The development of end-stage HF often involves an initial insult to the myocardium that reduces cardiac output and arterial baroreceptor stimulation leading to a compensatory increase in sympathetic nervous system activity, which ultimately results in cardiac dysfunction and remodeling [2], [3] In fact, sympathetic hyperactivity is associated with poor prognosis and constitutes an independent predictor of mortality [4], [5]. For counteracting sympathetic hyperactivity, the use of β-blockers is mandatory for HF therapy [6].

The treatment with β-blockers decreases sympathetic activity measured directly by microneurography performed on the anterior fibular nerve of HF patients [7], and promotes positive impact on cardiac function associated with a reverse remodeling [8]. In a genetic model of HF based on disruption of α_2A_/α_2C_-adrenergic receptors from mouse genome, we have previously observed that the third generation β–blocker, carvedilol, has no impact on exercise capacity but display an anti-cardiac remodeling effect and improves cardiac contractility [9], [10], which is independent of changes in isolated cardiac myocyte Ca^2+^ transients [9]. Accumulated evidence shows that aerobic exercise training is also an important strategy for the prevention and treatment of cardiovascular diseases [11], besides being an efficient adjuvant therapy for HF. Aerobic exercise training improves exercise tolerance and cardiac contractility; the later associated with changes in cardiac Ca^2+^ handling [12]–[14].

To increase the knowledge about different impact of β-blockers and aerobic exercise training on cardiac and skeletal muscle, we previously compared the isolated effects of exercise training and carvedilol treatment on exercise tolerance and cardiac contractility and remodeling in mice with an early stage HF induced by sympathetic hyperactivity [13], [15]. We observed that both aerobic exercise training and carvedilol therapy improved, to the same extent, the ventricular function in mild HF. However, while the benefits of aerobic exercise training were mainly associated with increased aerobic capacity and capillary density of skeletal muscle, the benefits of carvedilol were restricted to the effect on cardiac structure [15]. Although both carvedilol and aerobic exercise training have been highly recommended to the treatment of HF, it is unknown whether the combination of aerobic exercise training and carvedilol has integrative effects on the treatment of HF. In addition, the cellular basis of associative therapy on cardiac contractility has not been clarified yet.

In the present study, we used a genetic model of sympathetic hyperactivity-induced HF in mice to determine the combined effects of carvedilol and aerobic exercise training on cardiac structure and function, and overall functional capacity. Furthermore, we studied the expression of proteins involved in cardiac intracellular Ca^2+^ regulation and Ca^2+^ transients isolated from cardiomyocytes of all mice studied.

## Materials and Methods

### Animal Care

**Figure 1:**
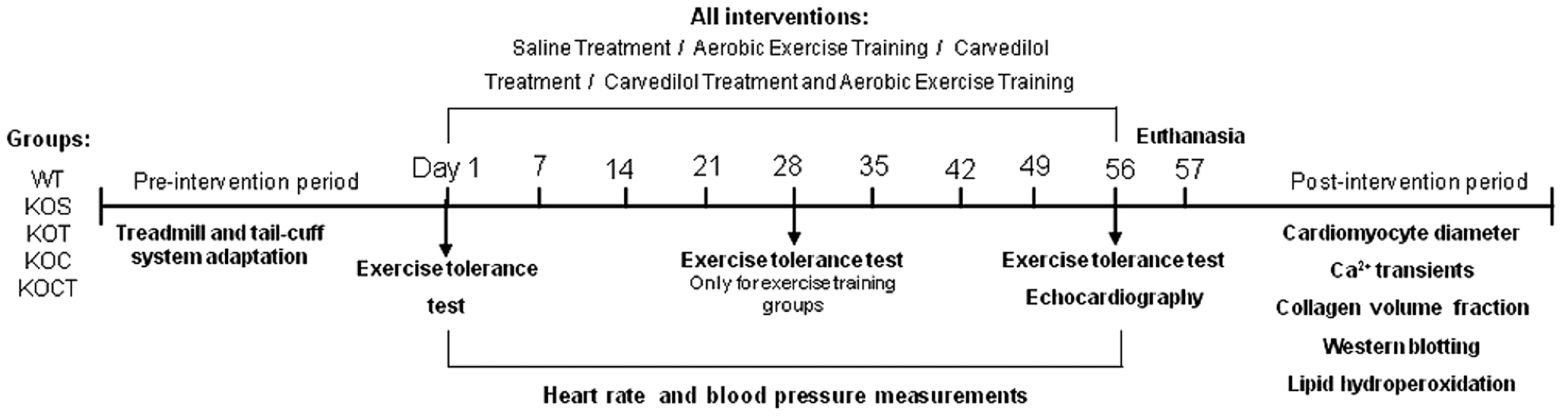
Experimental design. WT, wild type mice (control group); KOS, heart failure placebo; KOT, heart failure exercise trained; KOC, heart failure carvedilol-treated; and, KOCT, heart failure carvedilol-treated and exercise trained mice.

A cohort of male wild-type (WT) and congenic α_2A_/α_2C_ARKO mice (KO) with C57Bl6/J genetic background aged 5–7 months was studied. At this age, KO mice display advanced stage cardiomyopathy as previously described [16]. Genotypes were determined by PCR on genomic DNA obtained from tail biopsies using primers to detect the intact and disrupted genes. Mice were maintained in a light-controlled (12-hour light/dark cycle) and temperature-controlled (22°C) environment and were fed a pellet rodent diet (Nuvital Nutrientes S/A, Curitiba, PR Brazil) ad libitum and had free access to water. Mice were randomly assigned into five groups: control (WT), HF placebo (KOS), HF exercise trained (KOT), HF carvedilol-treated (KOC) and, HF exercise trained and carvedilol-treated (KOCT). This study was carried out in accordance with Ethical Principles of animal research adopted by the Brazilian College of Animal Experimentation (www.cobea.org.br). In addition, this study was approved by the Faculty of Medicine of University of São Paulo Ethics Committee (CEP 897/06). The experimental design is shown in Figure 1.

### Measurements and Procedures

#### Drug treatment and exercise training protocol

Drug treatment consisted of 8 weeks of placebo (saline) or carvedilol (38 mg/kg, Baldacci S.A., SP, Brazil) by gavage. Carvedilol is a third generation non-selective β-blocker with α_1_-blocking properties with a wide use in HF pharmacological therapy. Carvedilol did not change cardiac function, structure, Ca^2+^ or cardiac oxidative index in control mice. Therefore, we used only one WT group for further comparisons with KO groups. The dose of carvedilol was optimized to achieve comparable heart rate (HR) levels observed in age matched control group.

Moderate intensity aerobic exercise training was performed on a motor treadmill over 8 weeks, 5 days/week. The running speed and duration of exercise were progressively increased to elicit 60% of maximal speed, achieved during a graded treadmill exercise protocol, for 60 min. At the fourth week of exercise training, graded exercise test were repeated for readjusting the running speed. This intensity was maintained during the rest of the training period. All untrained mice were exposed to treadmill exercise (5 min) three times a week to become accustomed to exercise protocol and handling. The training sessions were performed during the dark cycle of the mice.

#### Graded treadmill exercise test

Exercise capacity, estimated by total distance run, was evaluated using a graded treadmill exercise protocol for mice. After being adapted to treadmill exercises over 1 week (10 min of exercise session), mice were placed on the treadmill streak and allowed to acclimatize for at least 30 min. Exercise began at 6 m/min with no grade and increased by 3 m/min every 3 min thereafter until exhaustion. The graded treadmill exercise test was performed in WT and KO mice before and after the aerobic exercise training period. Additionally, it was repeated at the fourth week of exercise training in order to adjust the training intensity as described above.

#### Cardiovascular measurements

HR and blood pressure were determined noninvasively using a computerized tail-cuff system (BP 2000 Visitech Systems, USA) described elsewhere [17]. Mice were acclimatized to the apparatus during daily sessions over 6 days, 1 week before starting the experimental period. HR measurements were obtained serially in WT and KO mice once a week throughout the 8 weeks of experiments. Noninvasive cardiac function was assessed by two-dimensional guided M-mode echocardiography, in halothane-anesthetized WT and HF mice (Fig. 1). Briefly, mice were positioned in the supine position with front paws wide open, and an ultrasound transmission gel was applied to the precordium. Transthoracic echocardiography was performed using a Sequoia 512 echocardiography machine (Acuson, Mountain View, CA, USA) equipped with a 14-MHz linear transducer and heart rate was kept similar in all groups studied during the evaluation to avoid artifactual changes in fractional shortening. Left ventricle systolic function was estimated by fractional shortening as follows:

Fractional shortening (%) = [(LVEDD – LVESD)/LVEDD] × 100, where LVEDD means left ventricular end-diastolic dimension, and LVESD means left ventricular end-systolic dimension.

For cardiac structural analysis, at the end of intervention period (Fig. 1), the heart was stopped at diastole (KCl, 14 mM) and dissected to obtain the left ventricle, which corresponds to the remaining organ upon removal of both atria and free wall of the right ventricle. For morphometric analysis, left ventricle samples obtained from the free wall, at the level of papillary muscle, were fixed in 4% buffered formalin and embedded in paraffin, cut in 4 µm sections and subsequently stained with hematoxylin and eosin. Two randomly selected sections from each animal were visualized by light microscopy using an objective with a calibrated magnification (400×). Myocytes with visible nucleus and intact cellular membranes were chosen for diameter determination. The width of individually isolated cardiomyocyte displayed on a viewing screen was manually traced, across the middle of the nuclei, with a digitizing pad and determined by a computer assisted image analysis system (Quantimet 520; Cambridge Instruments, UK). For each animal approximately 15 visual fields were analyzed. Quantification of left ventricular fibrosis was achieved by Sirius red staining. Two randomly selected sections from each animal were visualized by light microscopy using an objective with a calibrated magnification of 200×. Interstitial collagen area was quantified by a computer assisted image analysis system (Quantimet 520; Cambridge Instruments, UK). For each animal approximately five visual fields were analyzed.

#### Cardiomyocyte isolation and Ca^2+^ recording

To verify the cardiomyocyte Ca^2+^ transients, the other part of the animals received an intraperitoneal injection of pentobarbital sodium (100 mg/kg), and after full anesthesia the heart was rapidly removed. Cardiac ventricular myocytes were isolated, and imaged for [Ca^2+^]i as previously described [18]. The Ca^2+^ level measured with confocal microscopy was reported as F/F0, where F0 is the resting Ca^2+^ fluorescence. Images were obtained using the ZEISS Meta confocal microscope from CEMEL (Biological Sciences Institute, UFMG, Brazil).

#### Antibodies

Mouse monoclonal antibodies to SERCA2 (1∶2,500), Phospholamban (PLN, 1∶500) and Na^+^–Ca^2+^ exchanger (NCX, 1∶2,000) were obtained from Affinity BioReagents (Golden, CO, USA); rabbit polyclonal antibody to protein phosphatase type 1 (PP1, 1∶1,000) were obtained from Upstate (Lake Placid, NY, USA); phospho-Ser^16^-PLN (1∶5,000) and phospho-Thr^17^-PLN (1∶5,000) by Badrilla (Leeds, UK); Ryanodine antibody (RyR) was obtained from ABR Incorporation, EUA (1∶5,000); Glyceraldehyde-3-phosphate dehydrogenase (GAPDH, 1∶2,000) was obtained from Advanced Immunochemical (Long Beach, CA, USA). Targeted bands were normalized to cardiac GAPDH.

#### Western blot analysis

Left ventricular homogenates were analyzed by Western blotting to compare SERCA2, PLN, phospho-Ser^16^-PLN, phospho-Thr^17^-PLN, NCX, PP1 and RyR. Briefly, liquid nitrogen-frozen ventricles isolated from WT and HF mice were homogenized in a buffer containing 50 mM potassium phosphate buffer (pH 7.0), 0.3 M sucrose, 0.5 mM DTT, 1 mM EDTA (pH 8.0), 0.3 mM PMSF, 10 mM NaF, and phosphatase inhibitor cocktail (1∶100, Sigma-Aldrich; Saint Louis, MO). Samples were subjected to SDS-PAGE in polyacrylamide gels (6% or 10% depending on protein molecular weight). After electrophoresis, proteins were electrotransferred to nitrocellulose membrane (Amersham Biosciences; Piscataway, NJ, USA). Equal loading of samples (50 mg) and even transfer efficiency were monitored with the use of 0.5% Ponceau S staining of the blot membrane. The blotted membrane was then blocked (5% nonfat dry milk, 10 mM Tris-HCl, pH 7.6, 150 mM NaCl, and 0.1% Tween 20) for 2 h at room temperature and incubated with specific antibodies overnight at 4°C. Binding of the primary antibody was detected with the use of peroxidase-conjugated secondary antibodies (rabbit or mouse depending on the protein, 1∶10,000, for 1 h:30 min at room temperature) and developed using enhanced chemiluminescence (Amersham Biosciences, USA) detected by autoradiography. Quantification analysis of blots was performed with the use of Scion Image software (Scion based on NIH image).

#### Lipid hydroperoxides

Myocardial lipid hydroperoxide measurement was evaluated as an index of cardiac oxidative injury by the ferrous oxidation-xylenol orange technique (FOX2) [19]. Heart samples were homogenized (1∶20 w/v) in cold phosphate-buffered saline (100 mM, pH 7.4) and immediately centrifuged at 12,000 g for 20 min at 4°C.

Proteins were precipitated with trichloroacetic acid (10% w/v) and supernatant was mixed with FOX reagent and incubated for 30 min. The absorbance of the sample was read at 560 nm.

### Statistical Analysis

All variables showed normal distribution, when analyzed using the Shapiro-Wilk normality test, and therefore, the parametric statistical analysis was used. Data were expressed as mean ± standard error. The variables (peak of Ca^2+^ transient, decay Ca^2+^ transient, fractional shortening, cardiomyocyte diameter, collagen fraction and western blot analysis) were compared among groups by one-way analysis of variance (ANOVA) or one way ANOVA with repeated measures (exercise tolerance, heart rate, and blood pressure). For mortality rate, log rank analysis was used (Gehan-Breslow-Wilcoxon Test). In case of statistical significance, Tukey’s post hoc test was adopted. For all analyses, we adopted the significance level of P<0.05. The software used for statistical analysis was Statistica version 7.0.

## Results

### Effects of Therapies in Heart Rate and Blood Pressure

**Figure 2:**
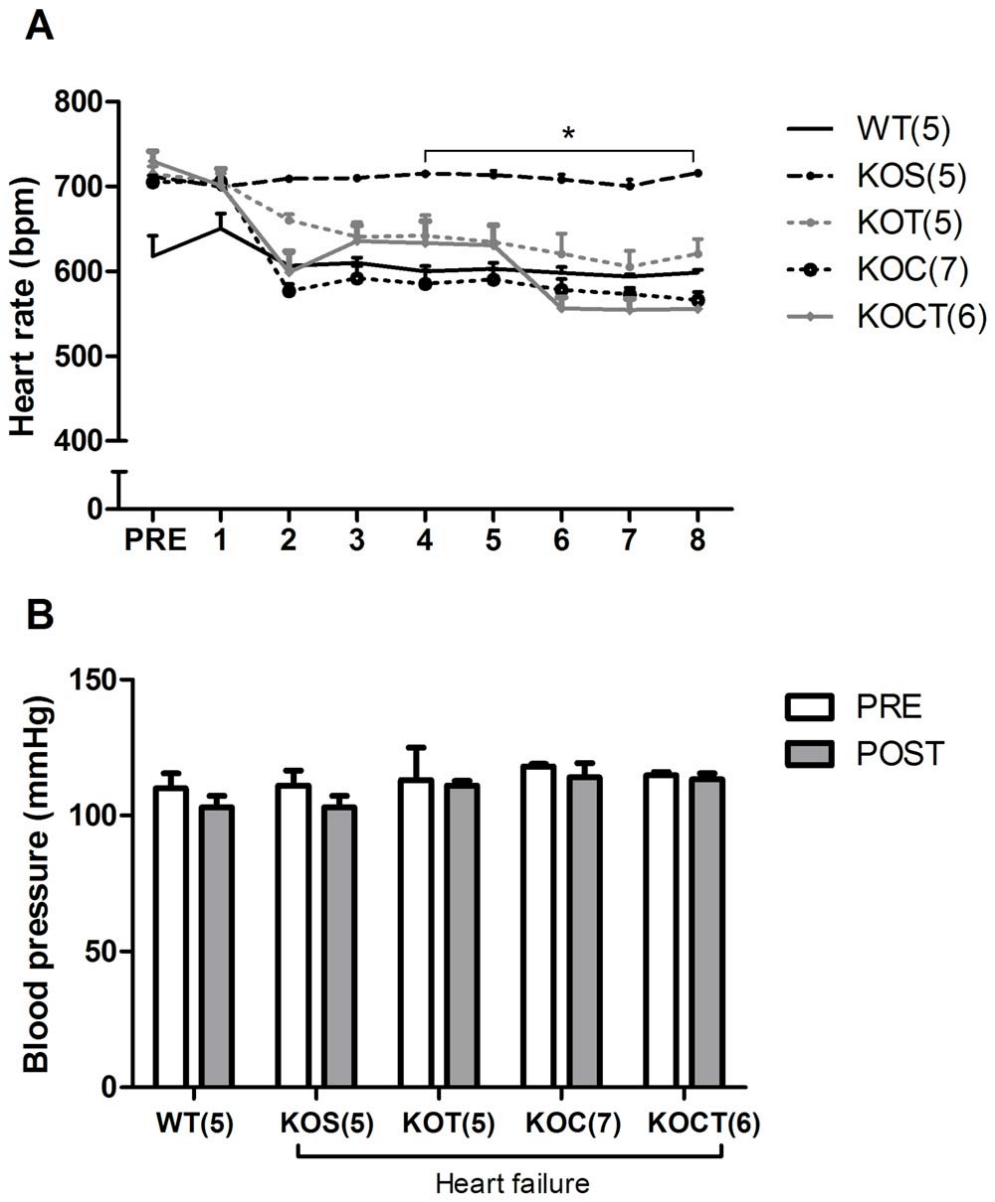
Heart rate (A) and blood pressure (B) during interventions in control (WT), heart failure placebo (KOS), heart failure exercise trained (KOT), heart failure carvedilol-treated (KOC) and, heart failure carvedilol-treated and exercise-trained (KOCT) mice. Note that all interventions decreased to the same extent the baseline HR of KO mice, which became similar to HR of WT group. Data are presented as mean ± SE. The number of animals studied is shown between parentheses (Panel A) or indicated by numerals on the abscissa (Panel B). *P<0.05 vs. other groups (groups indicated by lines).

KOS displayed baseline tachycardia when compared with age-matched control mice even though resting blood pressure was similar among all groups (P<0.05, Figures 2A and 2B). Blood pressure remained unchanged while baseline HR was reduced in WT group levels from the fourth week in both isolated and combined carvedilol and aerobic exercise training therapies, showing similar effectiveness of the therapies to reduce HR in KO toward WT levels (P<0.05, Figures 2A and 2B).

### Effects of Therapies on Survival, Exercise Tolerance, and Cardiac Function

**Figure 3:**
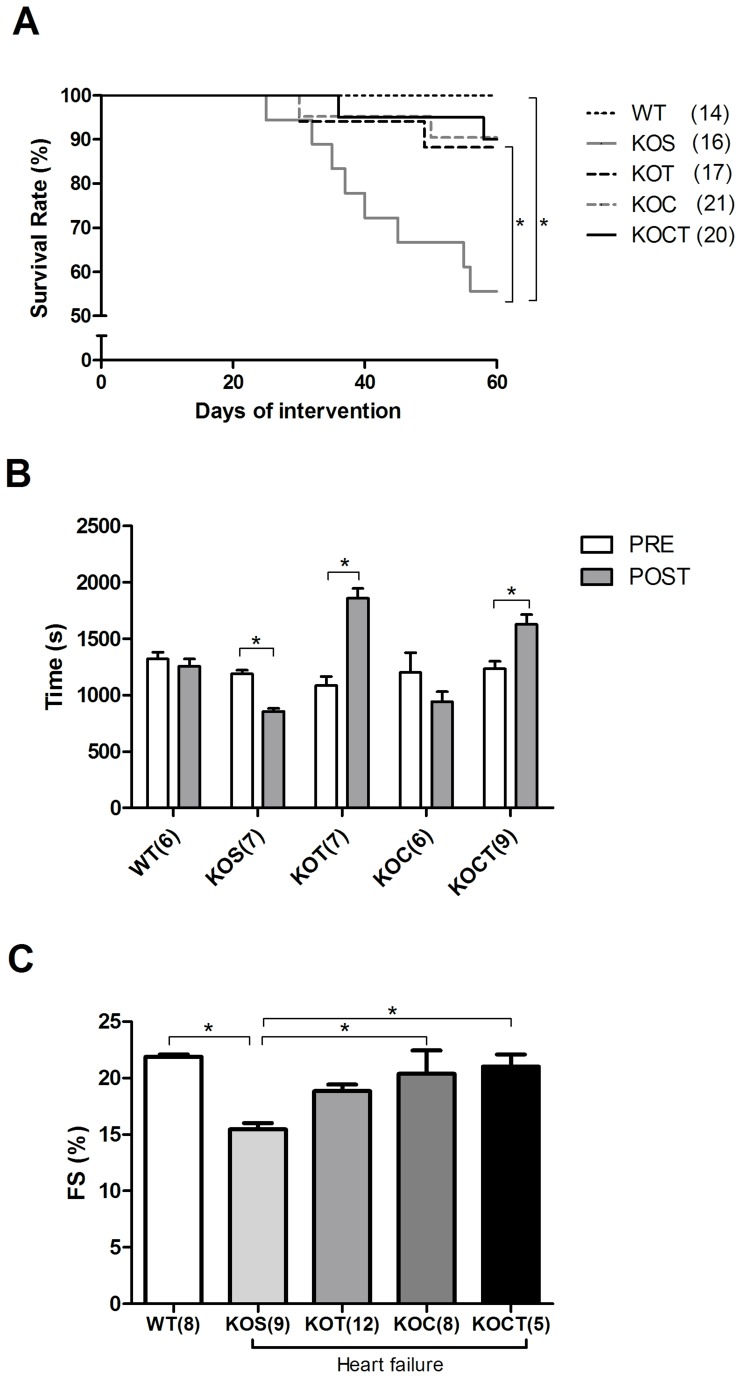
Survival (A), Exercise tolerance (B) and fractional shortening (FS, C) in control (WT), heart failure placebo (KOS), heart failure exercise trained (KOT), heart failure carvedilol-treated (KOC) and, heart failure carvedilol-treated and exercise trained (KOCT) mice. FS was evaluated after 8 weeks of intervention. Note that all interventions reduced mortality rate. However, only trained groups (KOT and KOCT) increased exercise tolerance. Data are presented as mean ± SE. The number of animals studied is shown between parentheses (Panel A) or indicated by numerals on the abscissa (Panels B and C). Panel A: *P<0.05 between KOS and WT, and among KOS and other groups. Panel B and C: *P<0.05 among groups indicated by lines.

While KOS mice presented 40% mortality rate after eight weeks of the study, aerobic exercise training, carvedilol treatment or carvedilol associated with exercise training significantly reduced HF mice mortality to 20%, 19% and 13%, respectively (P<0.05, Figure 3A). Exercise tolerance was reduced in KOS after 8 weeks of the study (P<0.05, Figure 3B). Both isolated aerobic exercise training and combined aerobic exercise training and carvedilol increased exercise tolerance in KO mice (P<0.05, Figure 3B) and exercise performance was comparable to that achieved for WT trained mice (data not shown). As expected, fractional shortening was reduced in KOS mice, and both carvedilol treatment and carvedilol combined to aerobic exercise training increased fractional shortening to WT mice levels (P<0.05, Figure 3C). The result cannot be explained by differences within groups in HR, because there were no significant differences between the HR under anesthesia (474±11.8, 475±7.6, 471±16.5, 465±20, 467±18.8 bpm for WT, KOS, KOT, KOC and KOCT, respectively). Therefore, HR would not be expected to artifactually change the fractional shortening.

### Effects of Therapies in Cardiac Structure, Renal Mass and Lung Water Content

**Figure 4:**
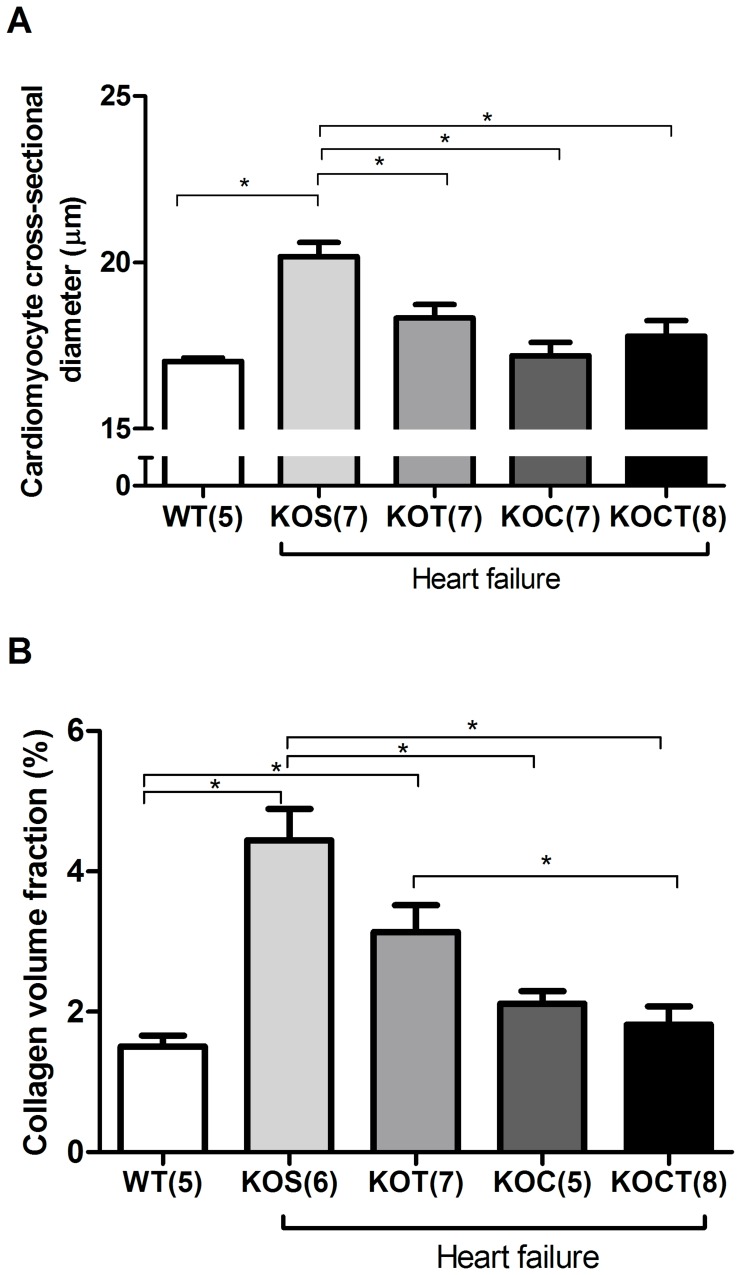
Cardiomyocytes cross-sectional area (A) and ventricular collagen volume fraction (B) in control (WT), heart failure placebo (KOS), heart failure exercise trained (KOT), heart failure carvedilol-treated (KOC) and, heart failure carvedilol-treated and exercise trained (KOCT) mice. Data are presented as mean ± SE. The number of animals studied is indicated by numerals on the abscissa. *P<0.05 among groups indicated by lines.

As expected, KOS mice displayed increased left ventricular mass (P<0.05, Table 1), cardiomyocyte cross sectional diameter and ventricular collagen volume fraction (P<0.05, Figure 4) compared with WT mice suggesting cardiac remodeling. All isolated and combined therapies were equally efficient in reducing left ventricular mass (P<0.05, Table 1) and cardiomyocyte cross sectional area in KOS mice to levels comparable to WT mice (P<0.05, Figure 4A). However, only carvedilol treatment and carvedilol combined to aerobic exercise training reduced the left ventricle collagen volume fraction to WT levels (P<0.05, Figure 4B).

**Table 1:** Body mass, cardiac chambers mass, kidney mass, lung mass and wet: dry lung mass ratio of control (WT), heart failure placebo (KOS), heart failure exercise trained (KOT), heart failure carvedilol-treated (KOC) and, heart failure carvedilol-treated and exercise-trained (KOCT).

	Body mass (g)		Right ventriclemass (g)	Left ventricle mass (g)	Kidney mass (g)	Lung mass (g)	Wet :dry lungmass (g)
WT		28±0.9 (10)	0.03±0.01 (10)	0.09±0.041 (10)	0.30±0.01 (6)	0.20±0.01 (9)	4.85±0.53 (8)
KOS		29±0.8 (7)	0.03±0.00 (7)	0.11±0.011*(7)	0.37±0.01*(4)	0.20±0.02(7)	6.18±0.67 (7)
KOT		29±1.3 (5)	0.03±0.001 (4)	0.09±0.001# (4)	0.37±0.01* (4)	0.17±0.01(5)	4.89±0.11 (5)
KOC		27±0.8 (8)	0.03±0.01 (8)	0.09±0.002# (8)	0.32±0.02^#^ ‡ (4)	0.19±0.01 (5)	4.85±0.21 (5)
KOCT		27±0.7 (12)	0.03±0.001 (12)	0.09±0.041# (12)	0.32±0.01^#^ ‡ (12)	0.26±0.02 (12)	4.76±0.20 (11)#

* P*<*0.05 vs. WT;

# P*<*0.05 vs. KOS;

‡ P<0.05 vs. KOT. The number of animals studied is shown between parentheses.

KOS mice displayed increased kidney mass compared with WT mice (P<0.05, Table 1) with no significant changes in plasma creatinine (data not shown). No differences were observed in lung mass and wet:dry lung mass between KOS and WT mice (Table 1). Combined carvedilol and aerobic exercise training, but not isolated therapies, significantly reduced kidney mass and wet:dry lung mass ratio when compared to KOS group (P<0.05, Table 1).

### Effects of Therapies in Cardiomyocyte Ca^2+^ Transients and Expression of Cardiac Ca^2+^ Handling Proteins

**Figure 5:**
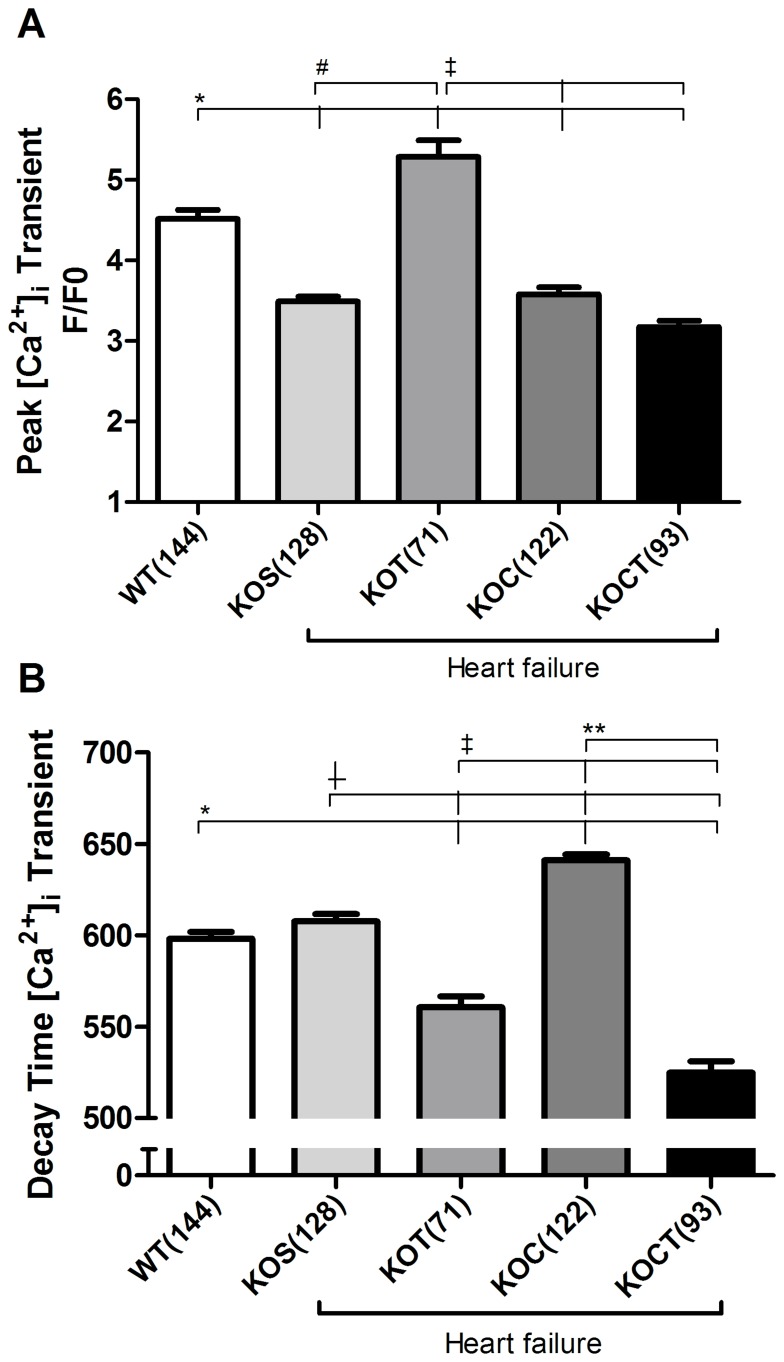
Intracellular Ca^2+^ transient in isolated ventricular myocytes in control (WT), heart failure placebo (KOS), heart failure exercise trained (KOT), heart failure carvedilol-treated (KOC) and, heart failure carvedilol-treated and exercise trained (KOCT) mice. A: Averaged data showing peak of Ca^2+^ transient B: Bar graph shows a comparison of Ca^2+^ transient kinetics (time from peak to 90% decay) between the different groups of cells. Data are presented as mean ± SE. The number of cells studied is indicated by numerals on the abscissa. *P<0.05 for WT vs. indicated groups; (WT vs. all groups in Panel A and WT vs. KOT, KOC and KOCT in Panel B); ^#^P<0.05 for KOS vs. KOT; ^+^P<0.05 for KOS vs. KOT, KOC and KOCT;^ ‡^P<0.05 for KOT vs. KOC and KOCT; **P<0.05 for KOC vs. KOCT.

**Figure 6:**
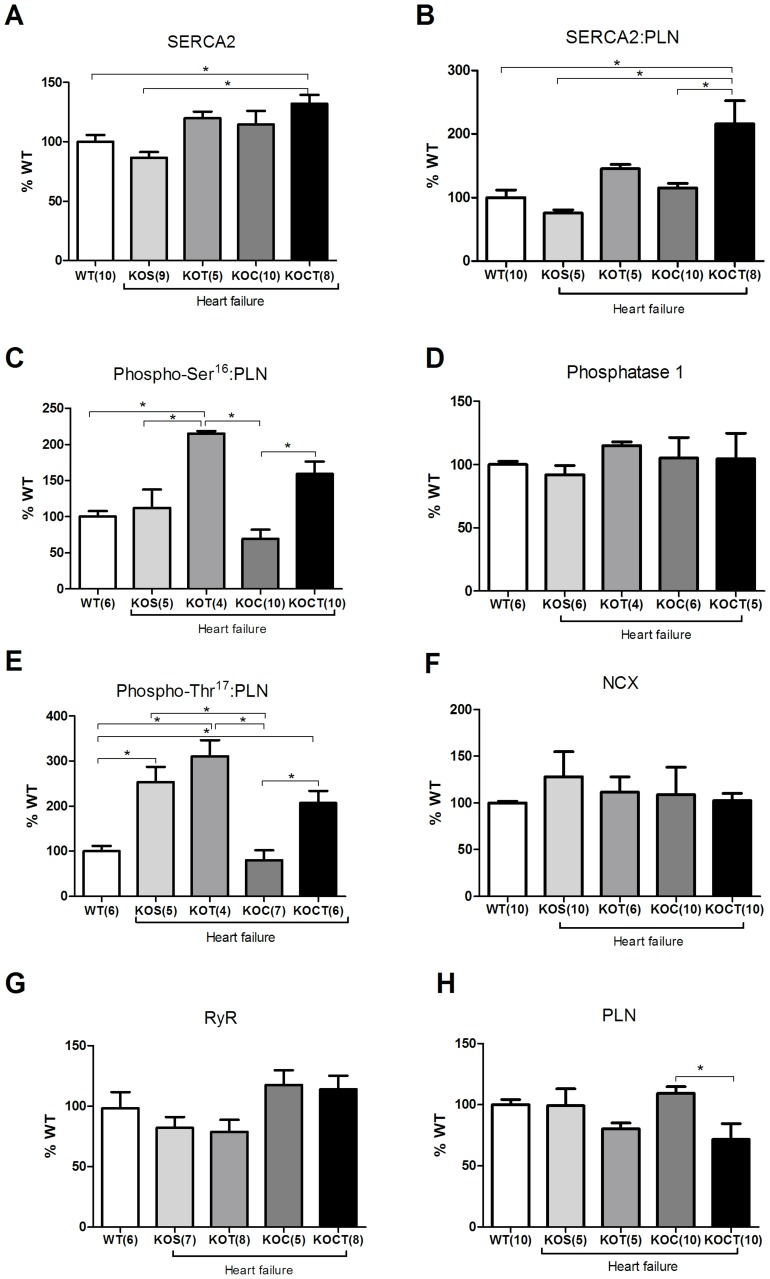
SERCA2 (A), SERCA2A:PLN (B), Phospho-Ser^16^-PLN:PLN (C), Phosphatase 1 (D), Phospho- Thr^17^-PLN:PLN (E), Na^+^– Ca^2+^ exchanger (NCX, F), Ryanodine receptor (RyR, G), Phospholamban (PLN, H). Data are presented as mean ± SE. *P<0.05 among groups indicated by lines.

Given the fact that Ca^2+^ handling is closely linked to cardiac contractile function regulation, we further examined the Ca^2+^ transients in isolated cardiomyocytes from all groups studied. Cardiomyocytes from KOS mice displayed reduced peak of Ca^2+^ transient compared with WT mice(P<0.05, Figure 5A). Aerobic exercise training increased peak of Ca^2+^ transient compared with cardiomyocytes from KOS mice (P<0.05, Figure 5A), which was not changed by isolated carvedilol treatment (Figure 5A). Surprisingly, carvedilol associated with aerobic exercise training had no impact on peak of Ca^2+^ transient (Figure 5A). Diastolic Ca^2+^ decay were not changed in KOS group when compared to WT group (Figure 5B), but increased by isolated carvedilol therapy (P<0.05, Figure 5B). Interestingly, both isolated aerobic exercise training and combined carvedilol and aerobic exercise training therapies decreased Ca^2+^ decay compared with KOS group (P<0.05, Figure 5B). To gain further insight into this response, we investigated the expression of key cardiac Ca^2+^ handling proteins. We observed that SERCA2 expression levels were not changed in KOS when compared to WT mice (Figure 6A). Interestingly, combined carvedilol and aerobic exercise training therapies significantly increased SERCA2 expression and SERCA2:PLN ratio compared with WT and KOS mice (P<0.05, Figures 5A and 5B). Phospho-Ser^16^-PLN:PLN ratio was increased by both aerobic exercise training or combined carvedilol and aerobic exercise training therapies in KO mice compared with WT and KOC, respectively (P<0.05, Figure 6C). The PP1 expression, which is mainly involved in dephosphorylating PLN at Ser^16^ residue, was similar among groups studied (Figure 6D). Phospho-Thr^17^-PLN:PLN ratio was significantly increased in KOS, KOT and KOCT compared with WT mice (P<0.05, Figure 6E). The KOC group presented similar phospho-Thr^17^-PLN:PLN levels to WT mice (Figure 6E).

No changes were observed in NCX, PLN and RyR protein expression among the groups studied (Figures 6F and 6G). GAPDH protein levels remained unchanged in all blots analyzed and among the four groups studied (data not shown) and were used to normalize the cardiac Ca^2+^ handling protein levels.

### Effects of Therapies on Marker of Oxidative Stress

**Figure 7:**
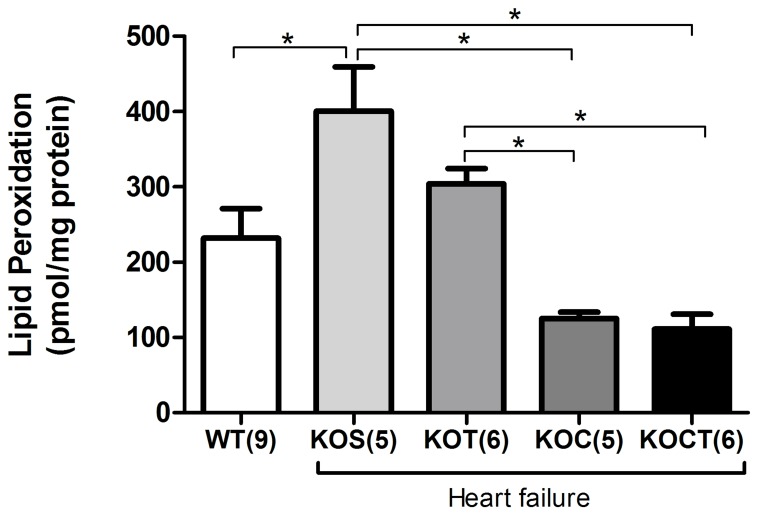
Lipid peroxidation in control (WT), heart failure placebo (KOS), heart failure exercise trained (KOT), heart failure carvedilol-treated (KOC) and, heart failure carvedilol-treated and exercise trained (KOCT) mice. Data are presented as mean ± SE. The number of animals studied is indicated by numerals on the abscissa. *P<0.05 among groups indicated by lines.

As expected, KOS mice displayed significantly increased cardiac lipid peroxides compared with WT mice (P<0.05, Figure 7). Carvedilol treatment or combined carvedilol and aerobic exercise training therapies reduced lipid peroxidation compared with KOS group (P<0.05, Figure 7), while isolated aerobic exercise training therapy had no impact on cardiac lipid peroxides compared with KOS group (Figure 7).

## Discussion

Here, we report that combined carvedilol and aerobic exercise training therapies integrate the distinctly different beneficial effects of isolated therapies on exercise capacity, ventricular function and remodeling associated with improved Ca^2+^ homeostasis and reduced ventricular oxidative stress. The main findings of the present study are that combined carvedilol and aerobic exercise training therapies: a) reduce mortality toward WT mice group level, b) improve exercise tolerance, c) re-establish left ventricle contractility and leads to a ventricular reverse remodeling, d) alter expression levels of proteins involved in Ca^2+^ handling and, e) prevent ventricular lipid peroxidation.

In the present study, we observed that improved exercise tolerance was restricted to exercised groups while re-established ventricular contractility and anti-cardiac remodeling were mainly observed in the groups receiving carvedilol. It is well-known that increased exercise tolerance, improved ventricular function and reduced cardiac remodeling are associated with improved survival in cardiovascular disease [20]–[22].

Presently, both isolated aerobic exercise training and carvedilol therapies improved survival, which might be related to their specific effects on exercise tolerance (KOT group) and ventricular function/remodeling, respectively. Therefore, our data suggest that aerobic exercise training and β-blockers have a positive impact on HF by different mechanisms and these findings corroborate previous studies from our group [9], [13]–[15] and others [7], [23], [24]. Of great interest, here we demonstrated that the positive effect of isolated therapies on survival, exercise tolerance and cardiac contractility and remodeling are preserved and integrated when aerobic exercise training is associated to carvedilol therapy.

Regarding the molecular mechanisms underlying the positive effects of isolated therapies, we have previously provided compelling evidence that aerobic exercise training improves cardiac Ca^2+^ handling [13], [14], which is directly associated with cardiac contractility. In fact, we presently observed that aerobic exercise training improves cardiac Ca^2+^ homeostasis by increasing the peak of Ca^2+^ transient, decreasing diastolic Ca^2+^ decay associated with increased phosphorylation of PLN at Ser^16^ and Thr^17^ in KO mice. Considering that phosphorylation of PLN at either Ser^16^ or Thr^17^ removes the inhibitory effect of PLN on SERCA2, it is reasonable to suggest that increased levels of phosphorylated PLN induced by aerobic exercise training contribute to the improved Ca^2+^ reuptake, as reported by faster diastolic Ca^2+^ decay. Carvedilol treatment had no impact on Ca^2+^ transient dynamics, since it failed to increase Ca^2+^ peak and prolonged Ca^2+^ decay, and significantly attenuated phospho-Ser^16^-PLN and Ser^17^-PLN expression levels. We previously demonstrated that despite no impact on Ca^2+^ transients, carvedilol reduces phosphorylation of troponin I at Ser^23/24^-residues [9], which ultimately leads to an increased sensitivity of contractile myofilament to Ca^2+^ associated with the antioxidant properties of carvedilol. In fact, carvedilol has a well-recognized antioxidant activity [25]–[27], which led us to investigate whether it would reduce myocardial oxidative stress in KO groups. Indeed, only groups treated with carvedilol reduced ventricular oxidative stress.

The molecular effects of combined carvedilol and aerobic exercise training therapies also rely on the integrative effects of isolated therapies. Combined therapies increased SERCA2 and phosphorylation of PLN expression at both Ser^16^ and Thr^17^ residues and reduced myocardial oxidative stress. Interestingly, these responses reflected on faster Ca^2+^ decay time observed in KOCT compared with KOS, KOT and KOC groups, which suggests a synergistic action of combined therapies on ventricular relaxation. Therefore, we provide compelling evidence that the combination of carvedilol and aerobic exercise training may represent better prognostic power in life long-treatment. It will be important, however, to further explore whether life long-term combination therapy leads to increased efficacy in reducing mortality and if so, what is the potential role of the preferential Ca^2+^ transient improvement associated to aerobic exercise training versus the greater ventricular anti-remodeling and antioxidant effects mediated by carvedilol.

The relative contribution of aerobic exercise training and carvedilol for improving cardiac function and survival is hampered by known limitations in clinical studies including patients number, etiology, co-morbidities and genetic makeup to name a few. In the present study, we took advantage of a congenic genetic model of sympathetic hyperactivity-induced HF to assess potential differences in cardiac structure and function when the animals were treated with carvedilol or exercised at a moderate level to achieve comparable HR exhibited by the WT group for two months. Therefore, the isolated effect of each therapy could be considered equally efficient in reducing HR, an important marker of both aerobic exercise training and β-blocker therapy efficacy.

### Conclusion

Taken together, we provided evidence that combined therapies with carvedilol and aerobic exercise training integrate the beneficial effects of isolated ones on survival, exercise tolerance and cardiac contractility and structure. The molecular mechanisms underlying the beneficial effects of combined therapies rely on the improved cardiac Ca^2+^ homeostasis mainly related to moderate aerobic exercise training effect and reduced myocardial oxidative stress and reverse ventricular remodeling associated with carvedilol therapy.
