# Correction: Integrative Effect of Carvedilol and Aerobic Exercise Training Therapies on Improving Cardiac Contractility and Remodeling in Heart Failure Mice

**DOI:** 10.1371/annotation/ae03273e-5ed4-4c8f-a985-b39503b2e7e5

**Published:** 2013-06-28

**Authors:** Andréa S. Vanzelli, Alessandra Medeiros, Natale Rolim, Jan B. Bartholomeu, Telma F. Cunha, Luiz G. Bechara, Enéas R. M. Gomes, Katt C. Mattos, Raquel Sirvente, Vera Maria Cury Salemi, Charles Mady, Carlos E. Negrao, Silvia Guatimosim, Patricia C. Brum

The name of the tenth author was given incompletely. The correct name is: Vera Maria Cury Salemi. The correct abbreviation in Contributions is: VMCS. 

