# Correction: Integrative Effect of Carvedilol and Aerobic Exercise Training Therapies on Improving Cardiac Contractility and Remodeling in Heart Failure Mice

**DOI:** 10.1371/annotation/f82c53c5-e15c-470c-b5e7-7732e1435cda

**Published:** 2013-07-01

**Authors:** Andréa S. Vanzelli, Alessandra Medeiros, Natale Rolim, Jan B. Bartholomeu, Telma F. Cunha, Luiz G. Bechara, Enéas R. M. Gomes, Katt C. Mattos, Raquel Sirvente, Vera Salemi, Charles Mady, Carlos E. Negrao, Silvia Guatimosim, Patricia C. Brum

The name of the sixth author was given incorrectly. The correct name is: Luiz R. G. Bechara. The correct abbreviation in Citations is: LRGB. 

